# Inflammatory Response in Preterm and Very Preterm Newborns with Sepsis

**DOI:** 10.1155/2016/6740827

**Published:** 2016-05-16

**Authors:** Enrique Segura-Cervantes, Javier Mancilla-Ramírez, Jorge González-Canudas, Erika Alba, René Santillán-Ballesteros, Deneb Morales-Barquet, Gabriela Sandoval-Plata, Norma Galindo-Sevilla

**Affiliations:** ^1^Departamento de Epidemiología, Instituto Nacional de Perinatología, Secretaría de Salud (SSa), Montes Urales 800, Colonia Lomas de Virreyes, 11000 Mexico City, Mexico; ^2^Escuela Superior de Medicina (ESM), Instituto Politécnico Nacional (IPN), Avenida Salvador Diaz Miron S/N, Esquina Plan de San Luis, Colonia Casco de Santo Tomás, Delegación Miguel Hidalgo, 11340 Mexico City, Mexico; ^3^Hospital de la Mujer, SSa, Prolongación Salvador Diaz Miron 374, Colonia Casco de Santo Tomás, Delegación Miguel Hidalgo, 11340 Mexico City, Mexico; ^4^Laboratorio Silanes, Amores 1304, Colonia del Valle, 03100 Mexico City, Mexico; ^5^Departamento de Infectología e Inmunología, Instituto Nacional de Perinatología, SSa, Montes Urales 800, Colonia Lomas de Virreyes, 11000 Mexico City, Mexico; ^6^UCIREN, Instituto Nacional de Perinatología, SSa, Montes Urales 800, Colonia Lomas de Virreyes, 11000 Mexico City, Mexico

## Abstract

The response of the adaptive immune system is usually less intense in premature neonates than term neonates. The primary objective of this study was to determine whether immunological parameters vary between preterm (PT) neonates (≥32 weeks of gestational age) and very preterm (VPT) neonates (<32 weeks of gestational age). A cross-sectional study was designed to prospectively follow PT and VPT neonates at risk of developing sepsis. Plasma concentrations of IFN-*γ*, TNF-*α*, IL-6, IL-4, and IL-10 were detected using flow cytometry. C-reactive protein (C-RP) and the complex SC5b-9 were detected in the plasma using commercial kits. A total of 83 patients were included. The laboratory results and clinical histories showed that 26 patients had sepsis; 14 were VPT, and 12 were PT. The levels of C-RP, SC5b-9 (innate immune response mediators), and IL-10 or IL-4 (anti-inflammatory cytokines) were elevated during sepsis in both groups. IFN-*γ*, TNF-*α*, and IL-6 (proinflammatory cytokines) were differentially elevated only in PT neonates. The VPT neonates with sepsis presented increases in C-RP, SC5b-9, and anti-inflammatory cytokines but not in proinflammatory cytokines, whereas PT neonates showed increases in all studied mediators of inflammation.

## 1. Introduction

Neonatal sepsis affects up to 20% of very low birth weight premature newborns, causing death in up to 18% of these newborns. The health of neonates can deteriorate quickly, and the newborns can develop septic shock and die before agent identification and antimicrobial sensitivity tests are ready. Because inflammation is an important event in the sepsis process, many studies have focused on measuring inflammatory mediators to permit an early diagnosis of sepsis [1–6]; however, systematic reviews [7] reveal that the sensitivity of sepsis diagnosis through inflammatory mediators varied, and additional studies are required. Importantly, the data are very scarce for preterm neonates, and relatively large discrepancies are reported amongst studies.

Discrepancies might originate from the fact that gestational age is usually not considered when the mediators of inflammation are analysed, even when the immune response in neonates is likely to be immature. For example, IL-6, IFN-*α*, and TNF-*α* production in monocytes and conventional dendritic cells (cDCs) of preterm neonates are attenuated after stimulation via Toll-like receptors (TLRs) [8].

Furthermore, it is important to consider that the immune system develops throughout the foetal period. Research regarding the immune system throughout foetal development indicates that the adaptive immune response in the preterm neonate exhibits more vulnerability because the T cells are limited by a lack of clonal expansion because of the absence of contact with the antigens. Therefore, at birth, immunological memory does not exist, and there is poor costimulatory receptor expression, which results in poor lymphocyte stimulation with low cytokine and antibody production. The limitations of the adaptive immune system are more marked in premature newborns than in neonates at term [9, 10]. Ontogeny mechanisms have shown that T*γ*/*δ* and mature *β*-1a receptors predominate in neonates at <32 weeks of gestational age compared with term neonates who express T*α*/*β* and *β*-1b receptors on their lymphocyte cells [11]. The differential predominance of the lymphocyte populations expressing receptors with lower antigenic recognition abilities could account for the impaired adaptive immune response in premature newborns. The innate immune system of neonates is hindered compared with that of adults because of differences in their components; however, differences between neonates of different gestational ages are not expected. This study investigated the innate and adaptive immune response mediators in neonates of different gestational ages (GAs) who were at risk of developing sepsis.

## 2. Methods

### 2.1. Study Design

**Figure 1:**
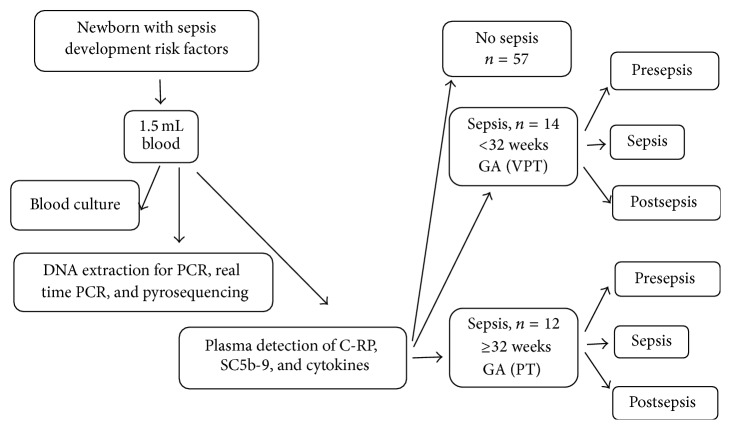
Flow diagram for patient selection.

This study sought to determine whether a differential inflammatory response exists between preterm (PT; >32 weeks of gestational age) and very preterm (VPT; ≤32 weeks of gestational age) neonates at risk of developing sepsis. Thus, a cross-sectional study was designed to follow neonates of mothers with chorioamnionitis, urinary infection,* Streptococcus* group B colonisation, and fever during labour or prolonged premature rupture of membranes; consent from the newborns' parents was required to participate in the study. Infants at the neonatal intensive care unit (NICU) were also included when they showed three or more clinical signs of sepsis such as apnoea, tachypnoea (>60/min), nasal flaring, retraction, cyanosis or respiratory distress-bradycardia (<100/min), tachycardia (>180/min), hypotonia, seizures, poor skin colour, irritability, or lethargy. We excluded neonates with haemotransfusion, herpetiform dermatitis, atopic eczema, congenital malformations, and/or laboratory confirmed TORCH (*Toxoplasma*, other,* Rubella virus*,* Cytomegalovirus*, herpes simplex virus-II) infections or those receiving gamma globulin IV. All participants were born at the Instituto Nacional de Perinatología (INPer), Mexico City. The Human Investigation Committee of the INPer approved this study. Because neonates in the NICU are continuously monitored, the results were identified as* presepsis* if the sample was taken a week before clinical or proven sepsis,* sepsis* if the neonate had clinical or proven sepsis while being studied, including samples taken within a range of 2 days, and* postsepsis* if the sample was provided a week after clinical or proven sepsis (Figure 1). The diagnosis of clinical sepsis is considered present when neonates had systemic inflammatory response syndrome (SIRS) according to those criteria recommended for preterm infants [12] and proven sepsis when besides the SIRS there was a blood culture positive for pathogenic bacteria. Neonates who did not develop clinical or diagnosed sepsis were not included in the analysis because only one sample was taken from them. The cases were classified according to the infant's age at birth as <32 or ≥32 weeks of GA [13–16]. Early-onset sepsis was defined as the first sepsis episode occurring <3 days of life, and late-onset sepsis was defined as occurring ≥3 days [17], but less than 4 weeks of life.

### 2.2. Microorganism Detection

Blood cultures were performed at the microbiology laboratory using standard techniques via the automated vac+/alert system (Organon Teknika, Toronto, ON). Bacterial identification was performed using the Vitek automated system (bioMérieux, France).

### 2.3. C-Reactive Protein (C-RP)

C-RP levels were analysed using a Minineph*™* Plus kit (The Binding Site Group Ltd., Birmingham, UK).

### 2.4. Complement

The complex C5b-9, which is formed when complement is activated by any of the four routes, is then coupled to the S protein to form the soluble complex SC5b-9, which was detected in the serum using a commercial kit (Quidel, Mountain View, CA, USA) according to a standard enzyme-linked immunosorbent assay (ELISA) procedure. Colour development in the plates was read using a spectrophotometer (Synergy 2, BioTek Instruments, Winooski, VT, USA) at 405 nm.

### 2.5. Cytokine Detection

The concentrations of IL-4, IL-6, IL-10, IFN-*γ*, and TNF-*α* were detected simultaneously using a human cytometric bead array (CBA) kit from BD (Becton, Dickinson Immunocytometry Systems, San Jose, CA, USA). Fifty microliters of plasma or the provided standard cytokines was added to the premixed microbeads in 12 mm × 75 mm Falcon tubes (BD). After the addition of 50 *μ*L of a mixture of PE-conjugated antibodies against the cytokines, the mixture was incubated for 2 h in the dark at room temperature. This mixture was washed and centrifuged at 500 g for 5 min, and the pellet was resuspended in 300 *μ*L of wash buffer. A FACS Aria II (BD) was used to read the samples. A standard curve including 9 concentrations was created for each determination. A typical sample mixture included 10 *μ*L of capture beads, 50 *μ*L of plasma, and 50 *μ*L of the detector. The samples were mixed for 2 h in the dark and were then washed and read in the cytometer. The results were analysed using FACS Diva*™* BD software.

### 2.6. Statistical Analysis

The clinical characteristics of the participants are presented using descriptive statistics (mean ± standard deviation for continuous variables and proportions for qualitative variables). Individual data were compared to normal reference ranges using Student's *t*-test for independent samples at a 95% confidence level, the *χ* ^2^ test, or Fisher's exact test for categorical variables. Intragroup comparisons based on a one-way analysis of variance with repeated measures (ANOVA) were initially performed. When Levene's homogeneity of variance test indicated significant differences, the Friedman test was used. Statistical analyses were performed using XL-STAT 2013, version 5.06, for Windows (Addinsoft, New York, NY, USA).

## 3. Results

### 3.1. Newborn Characteristics

Eighty-three newborns who were at risk of developing sepsis or had a suspected infection were sampled immediately after birth. The laboratory results and clinical history showed that 26 neonates had sepsis; 12 of the infants were PT, and 14 were VPT. When the two sepsis groups were compared, the clinical variables birth weight and weeks of gestation differed (*P* < 0.05, Student's *t*-test, for both cases); the sex relationship was similar between the groups (*P* = 0.892, *χ* ^2^ test). Two deaths occurred, one in each sepsis group. Early-onset sepsis was observed in 6/12 PT neonates and in 6/14 VPT neonates (Table 1).

### 3.2. Aetiological Agent Identification

The blood culture was positive in 16/26 sepsis cases. The most frequent microorganisms were* Klebsiella pneumoniae* (*n* = 6),* Escherichia coli* (*n* = 4), followed by* Candida spp.* (*n* = 2),* Enterobacter spp.* (*n* = 2),* Staphylococcus aureus* (*n* = 1),* Staphylococcus spp.* coagulase-negative (*n* = 2), and group B* Streptococcus* (*n* = 1). No differences in the number of isolated microorganisms were found between the PT and VPT groups (*P* > 0.05, *χ* ^2^ test); however, the VPT group showed more infections with gram-positive microorganisms (*n* = 5) and only two cases with gram-negative microorganisms, whereas the PT group presented more frequent infections with gram-negative microorganisms (*n* = 8).

### 3.3. High Levels of Innate Immune Response Mediators during Sepsis in Neonates

#### 3.3.1. C-RP

**Figure 2:**
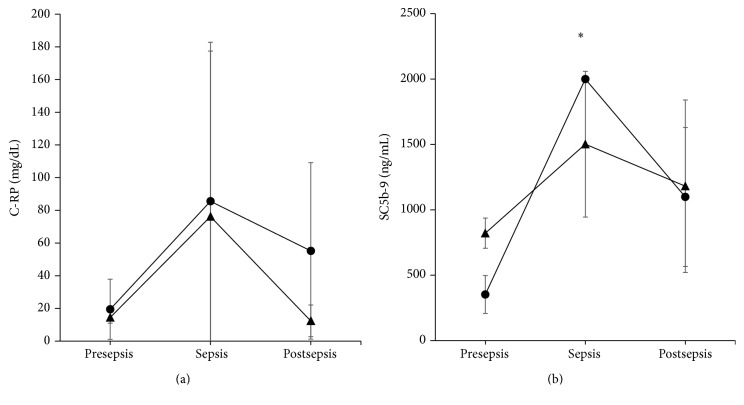
Innate immunity. C-RP (a) and active complement in the form of complex SC5b-9 (b) were both elevated at sepsis (mean ± standard deviation) in the PT (circle) or VPT (triangle) groups compared with presepsis and postsepsis. However, only PT neonates group was different for SC5b-9 (^*∗*^ *P* < 0.01, ANOVA).

This protein is synthesized by the liver in response to an infection as a part of the innate immunity. The mean (±standard deviation) values for C-RP were 19.5 ± 21.2, 85.57 ± 99.18, and 55.2 ± 60.36 mg/dL for presepsis, sepsis, and postsepsis, respectively, for PT newborns (*P* = 0.403, ANOVA); these values did not differ from the presepsis, sepsis, and postsepsis values of 14.5 ± 4.95, 76.2 ± 119.19, and 12.4 ± 10.83 mg/dL, respectively, for the VPT (*P* = 0.435, ANOVA). During sepsis, the values were similar for both PT and VPT of the sepsis points (*P* = 0.885, Student's *t*-test; Figure 2(a)).

#### 3.3.2. Complement

The values for active complement were similar to C-RP; they increased during sepsis independent of the gestational age of the neonates. The values for the PT group were 353 ± 177.15, 2,000 ± 0.86, and 1,098.5 ± 613.61 ng/mL, respectively (*P* = 0.002, ANOVA). For the VPT neonates, the values were 821.5 ± 163.34, 1,502 ± 643.59, and 1,181 ± 807.27 ng/mL (*P* = 0.515, ANOVA), respectively. No differences were observed between the PT and VPT groups during sepsis (*P* = 0.173, Student's *t*-test; Figure 2(b)).

### 3.4. High Levels of Proinflammatory Cytokines during Sepsis in PT and VPT Neonates

#### 3.4.1. IFN-*γ*

**Figure 3:**
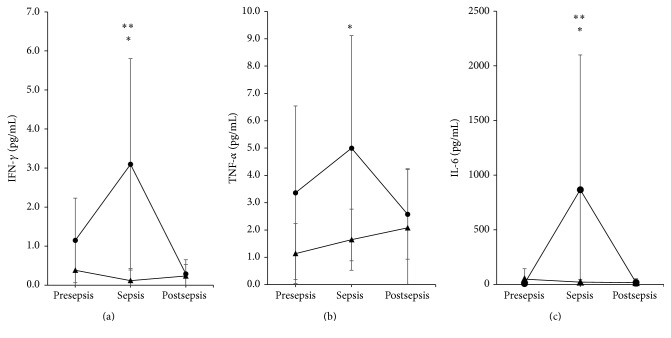
Proinflammatory cytokines. IFN-*γ* (a), TNF-*α* (b), and IL-6 (c) showed similar patterns during the sepsis process (mean ± standard deviation). During sepsis, the values for the PT infants (circle) were elevated compared with VPT (triangle) infants (^*∗*^ *P* < 0.01, Student's *t*-test). Values at sepsis were higher than pre- and postsepsis in the case of IFN-*γ* and IL-6 for PT group (^*∗∗*^ *P* < 0.01, Friedman test).

One week before the beginning of clinical sepsis (presepsis) or a week after clinical sepsis (postsepsis), the values for IFN-*γ* in the neonates were approximately normal. For the PT infants, the mean (±standard deviation) presepsis value was 1.147 ± 1.08 pg/mL, and the postsepsis mean was 0.289 ± 0.36 pg/mL. At clinical sepsis, the value was 3.095 ± 2.71 pg/mL (*P* = 0.004, Friedman test). The IFN-*γ* values remained high for approximately three to five days during clinical sepsis; however, the values for the VPT infants throughout the sepsis process were 0.381 ± 0.74 pg/mL, 0.117 ± 0.31 pg/mL, and 0.234 ± 0.30 pg/mL at presepsis, sepsis, and postsepsis, respectively (*P* = 0.186, Friedman test). Individual comparisons between both the PT and VPT neonates with regard to the IFN-*γ* values during sepsis showed significant differences (*P* < 0.01, Student's *t*-test; Figure 3(a)). Therefore, the values for IFN-*γ* varied depending on the time of sepsis and the gestational age of the newborn. This issue should be considered when this cytokine is used for sepsis diagnosis.

#### 3.4.2. TNF-*α*

The dynamic of TNF-*α* during the sepsis process of the neonates was similar to that of INF-*γ* and depended on the time of sepsis and the gestational age of the infant. The values for the PT neonates were 3.362 ± 3.18 pg/mL, 4.995 ± 4.12 pg/mL, and 2.574 ± 1.64 pg/mL at presepsis, sepsis, and postsepsis, respectively (*P* = 0.318, ANOVA). The presepsis, sepsis, and postsepsis values for the VPT neonates were 1.138 ± 1.10 pg/mL, 1.646 ± 1.12 pg/mL, and 2.079 ± 2.16 pg/mL, respectively (*P* = 0.452, ANOVA). The clinical sepsis times between the PT and VPT groups differed (*P* = 0.021, Student's *t*-test; Figure 3(b)).

#### 3.4.3. IL-6

The IL-6 values for the PT group were 8.39 ± 12.74 pg/mL, 866.64 ± 1233.11 pg/mL, and 17.6 ± 35.40 pg/mL at presepsis, sepsis, and postsepsis, respectively (*P* = 0.003, Friedman test). For the VPT group, the presepsis, sepsis, and postsepsis values were 48.58 ± 95.38 pg/mL, 21.5 ± 22.80 pg/mL, and 16.3 ± 25.56 pg/mL, respectively (*P* = 0.47, Friedman test). A difference was observed between PT and VPT groups with regard to the sepsis time points (*P* = 0.027, Student's *t*-test; Figure 3(c)).

### 3.5. High Levels of Anti-Inflammatory Cytokines Mediate Specific Immune Responses during Sepsis in Neonates

#### 3.5.1. IL-4

**Figure 4:**
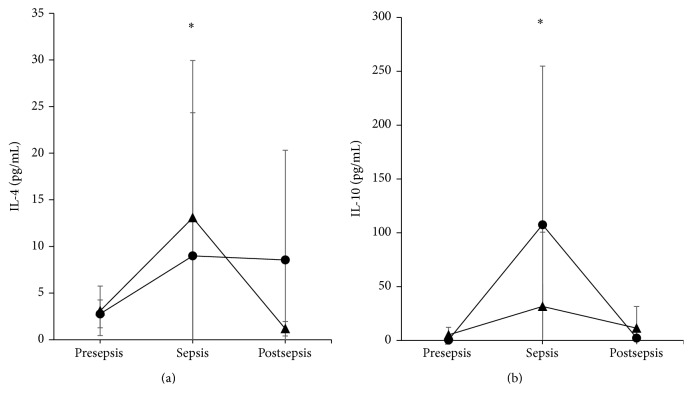
Anti-inflammatory cytokines. IL-4 (a) and IL-10 (b) increased in both the PT (circle) and VPT (triangle) groups during sepsis (mean ± standard deviation), compared to pre- and postsepsis. Only PT group was statistically different (^*∗*^ *P* < 0.01, Friedman test). At sepsis time no differences were found between PT and VPT groups.

The IL-4 graph displays a different dynamic than the aforementioned proinflammatory cytokines. The values for the PT group were 2.76 ± 1.50 pg/mL, 8.99 ± 15.35 pg/mL, and 8.55 ± 11.76 pg/mL at presepsis, sepsis, and postsepsis, respectively (*P* = 0.92, Friedman test). The values for the VPT group were 3.09 ± 2.65 pg/mL, 13.08 ± 16.85 pg/mL, and 1.17 ± 0.78 pg/mL at presepsis, sepsis, and postsepsis, respectively (*P* = 0.014, Friedman test). The values at the sepsis time point for the VPT group were slightly higher than those for the PT group, although no difference was detected (*P* = 0.527, Student's *t*-test; Figure 4(a)).

#### 3.5.2. IL-10

The IL-10 values for the PT group were 0.457 ± 0.60 pg/mL, 107.5 ± 147.40 pg/mL, and 2.25 ± 5.54 pg/mL at presepsis, sepsis, and postsepsis, respectively (*P* = 0.002, Friedman test). The values for the VPT group were 5.13 ± 7.14 pg/mL, 31.6 ± 68.90 pg/mL, and 11.37 ± 20.22 pg/mL at presepsis, sepsis, and postsepsis, respectively (*P* = 0.789, Friedman test). No differences were observed between the PT and VPT groups with regard to IL-10 at sepsis (*P* = 0.123, Student's *t*-test; Figure 4(b)).

### 3.6. Sepsis Identification

In summary, the identification of microorganisms using blood culture (Table 2) was possible in 16/26 sepsis cases and did not differ between the PT and VPT groups in the identified proportions (*P* = 0.62, *χ* ^2^ test). The type of isolated microorganism differed between the groups, with more frequent gram-positive microorganisms in the VPT group and more gram-negative microorganisms in the PT group (*P* = 0.003, *χ* ^2^ test). The immunological parameters were analysed as positive or negative with respect to the cut-off value. They were considered as positive when at least one innate immunity, proinflammatory, or anti-inflammatory cytokine result tested positive at sepsis. The innate immunity factors C-RP and active complement SC5b-9 were identified in 8/10 patients with sepsis in the PT group and in 5/5 patients with sepsis in the VPT group, resulting in 13/15 patients with sepsis identified. No differences were observed between groups (*P* > 0.05, *χ* ^2^ test). The proinflammatory cytokines IFN-*γ*, TNF-*α*, and IL-6 were elevated in 9/11 PT infants with sepsis but only in 1/12 VPT infants with sepsis (*P* < 0.05, *χ* ^2^ test), resulting in 10/23 infants with sepsis identified. The anti-inflammatory cytokines IL-4 and IL-10 were elevated in 8/11 PT infants with sepsis and in 7/12 VPT infants with sepsis (*P* > 0.05, *χ* ^2^ test), resulting in 15/23 identified infants with sepsis.

## 4. Discussion

Human studies [6, 18, 19] and animal models [20–22] have demonstrated that sepsis provokes a simultaneous release of both pro- and anti-inflammatory cytokines into the blood beginning at the onset of the disease; however, the literature reports conflicting results concerning the detection of inflammatory mediators in the diagnosis of sepsis for premature neonates [7]. As a consequence, the current cross-sectional study was designed to evaluate whether mediators that are released before, during, and after the sepsis processes present differentiable dynamics with regard to neonates who are older or younger than 32 weeks of gestational age because these groups exhibit different levels of immune system maturity due to foetal development [23]. This difference in immune system maturity could be the origin of the heterogeneity observed in the evaluation of the immune response during sepsis [7, 8]. The release of native and specific immune mediators was measured to have more elements to detect in the sepsis cases.

This study is the first to report that proinflammatory cytokine production in sepsis can differentially distinguish between the dynamics of the innate, proinflammatory, or anti-inflammatory response that are most likely related to the foetal development of the immune system. Here, neonates with sepsis had elevated levels of mediators of innate defences including C-RP and SC5b-9, independent of gestational age. This result is in agreement with the presepsin (another innate component recently studied) detection in neonates <32 weeks of GA [24].

During foetal development, the innate defences develop first. For example, granulocytes mature during the 12th week of gestation, monocytes/macrophages during the 16th week of gestation, and NK cells during the 21st week gestation; however, the adaptive defences, including T and B lymphocytes, appear first as immature forms beginning in the 7th week of gestation but are not yet ready to fully respond. The lymphocytes follow a maturation process that includes the differential expression of several surface molecules that provide unique characteristics and the abilities to tolerate “self-antigens” and to respond to external antigens. Lymphocytes are not fully mature until the 32nd gestational week. Before that, T lymphocytes lack the expression of the T*α*/*β* receptor but display predominant expression of the T*γ*/*δ* receptor, which is a receptor observed in mucosal immunity and has a limited repertoire for antigen recognition. Cytokine production differs between both types of cells [11]. Cytokines are the major inducible products of immune system cells. Because maturity is reached by the programed development of the foetus, the differential production of cytokines in organisms <32 weeks of gestation (VPT) might account for the differences in proinflammatory cytokine production compared with those at >32 weeks of gestation (PT). This situation was observed in the current work. The proinflammatory cytokines IFN-*γ*, TNF-*α*, and IL-6 were differentially elevated only in PT neonates but not in VPT neonates in whom the values were low. This finding is important and should be considered when understanding sepsis diagnoses based on proinflammatory cytokine production.

IL-4 was elevated in both groups; however, recent advances have shown that during T cell differentiation, IL-4 is predominantly produced by a subset of T*γ*/*δ* cell type 2 innate lymphoid cells (ILC2) that differentiate earlier than T*α*/*β* cells and could produce IL-4 before 32 gestational weeks of age [25].

IL-10 levels did not differ between the two groups. IL-10 is another cytokine that could be produced early during foetal development because it can be produced by B-1a cells [26], which is a precursor of B cells that differentiate during the 14th gestational week and predominate in the foetus until the 32nd week of GA [11].

The low response of the neonate immune cells has been additionally identified in animal models following* in vitro* stimulation. When monocytes and cDCs from preterm neonatal mice were stimulated to produce cytokines via TLR induction, a reduction in the levels of TNF-*α*, IFN-*α*, or IL-6 was observed. The authors also reported equivalent levels of IL-10 in both the preterm and at term groups [8].

The aetiological agent identification in this study coincides with other studies that identified agents for early- and late-onset sepsis [27]. Few cases reached gram-negative identification for VPT neonates; however, no differences in the cytokine production were elicited by gram-negative microorganisms (data not shown).

The measurement of the inflammatory mediators for sepsis diagnosis is extremely relevant for VPT neonates, who rarely provide plenty of blood for cultures. Thus, it is very important to distinguish the types of mediators that are candidates for sepsis diagnosis. SC5b-9 is a likely candidate because it increases in both PT and VPT neonates during sepsis. In addition, it is advantageous in that it can be detected in an under 1 : 10 dilution; this condition was recently used to measure cytokines in a mouse model of sepsis [28] and should be explored in humans.

## 5. Conclusions

The proinflammatory cytokines (TNF-*α*, IFN-*γ*, and IL-6) increase differentially in neonates with sepsis based on gestational age. As expected, these cytokine values were high for neonates ≥32 weeks of GA during sepsis; however, they were negligible for neonates born <32 weeks of GA. We therefore suggest that the interpretation of proinflammatory cytokines should consider the gestational age of the neonate. The levels of the anti-inflammatory cytokines IL-4 and IL-10 as well as the components of innate immunity C-RP and SC5b-9 were high during sepsis in both gestational age groups, unlike the proinflammatory cytokines.

## Figures and Tables

**Table 1:** Patients characteristics.

	PT sepsis *n* = 12	VPT sepsis *n* = 14	*P* value
Birth weight (g)	2068.6 ± 648	936.8 ± 200	>0.05^a^
Gestation weeks	34.56 ± 1.8	28.7 ± 1.3	>0.05^a^
Blood culture	8	8	>0.05^b^
Gender relationship (M/F)	4 : 1	4 : 3	>0.05^b^
Hospital stay (days)	18.5 ± 14	19.46 ± 14.4	<0.05^a^
Deaths	1	1	>0.05^b^
Early sepsis	6	6	>0.05^b^

^a^Student's *t*-test between the PT and VPT groups.

^b^ *χ* ^2^ test between the PT and VPT groups.

**Table 2:** Sepsis identification.

	PT	VPT	*P* value
Blood culture	8/12	8/14	>0.05
Microorganism (gram +/−)	1/8	5/2	<0.05
Innate immunity	6/7	5/5	>0.05
Proinflammatory cytokines	9/11	1/12	<0.05
Anti-inflammatory cytokines	7/11	6/12	>0.05

*χ* ^2^ test.

## References

[1] Hotoura E., Giapros V., Kostoula A., Spyrou P., Andronikou S. (2012). Pre-inflammatory mediators and lymphocyte subpopulations in preterm neonates with sepsis. *Inflammation*.

[2] Kurt A. N. C., Aygun A. D., Godekmerdan A., Kurt A., Dogan Y., Yilmaz E. (2007). Serum IL-1*β*, IL-6, IL-8, and TNF-*α* levels in early diagnosis and management of neonatal sepsis. *Mediators of Inflammation*.

[3] Ng P. C. (2004). Diagnostic markers of infection in neonates. *Archives of Disease in Childhood: Fetal and Neonatal Edition*.

[4] Ng P. C., Lam H. S. (2010). Biomarkers for Late-onset neonatal sepsis: cytokines and beyond. *Clinics in Perinatology*.

[5] Reis Machado J., Soave D. F., Da Silva M. V. (2014). Neonatal sepsis and inflammatory mediators. *Mediators of Inflammation*.

[6] Shah B. A., Padbury J. F. (2014). Neonatal sepsis an old problem with new insights. *Virulence*.

[7] Pammi M., Flores A., Leeflang M., Versalovic J. (2011). Molecular assays in the diagnosis of neonatal sepsis: a systematic review and meta-analysis. *Pediatrics*.

[8] Lavoie P. M., Huang Q., Jolette E. (2010). Profound lack of interleukin (IL)-12/IL-23p40 in neonates born early in gestation is associated with an increased risk of sepsis. *Journal of Infectious Diseases*.

[9] Wynn J., Cornell T. T., Wong H. R., Shanley T. P., Wheeler D. S. (2010). The host response to sepsis and developmental impact. *Pediatrics*.

[10] Futata E. A., Fusaro A. E., De Brito C. A., Sato M. N. (2012). The neonatal immune system: immunomodulation of infections in early life. *Expert Review of Anti-Infective Therapy*.

[11] Palmer A. C. (2011). Nutritionally mediated programming of the developing immune system. *Advances in Nutrition*.

[12] Wynn J. L., Wong H. R. (2010). Pathophysiology and treatment of septic shock in neonates. *Clinics in Perinatology*.

[13] Bochennek K., Fryns E., Wittekindt B. (2016). Immune cell subsets at birth may help to predict risk of late-onset sepsis and necrotizing enterocolitis in preterm infants. *Early Human Development*.

[14] Gibbons D. L., Haque S. F. Y., Silberzahn T. (2009). Neonates harbour highly active *γδ* T cells with selective impairments in preterm infants. *European Journal of Immunology*.

[15] Serwatowska-Bargiel A., Wasik M., Kornacka M. K., Górska E., Kozarski R. (2013). T-cell subpopulations *αβ* and *γδ* in cord blood of very preterm infants: the influence of intrauterine infection. *Achivum Immunologiae at Therapiae Experimentallis*.

[16] Matoba N., Yu N., Mestan K. (2009). Differential patterns of 27 cord blood immune biomarkers across gestational age. *Pediatrics*.

[17] Hornik C. P., Fort P., Clark R. H. (2012). Early and late onset sepsis in very-low-birth-weight infants from a large group of neonatal intensive care units. *Early Human Development*.

[18] Iskander K. N., Osuchowski M. F., Stearns-Kurosawa D. J. (2013). Sepsis: multiple abnormalities, heterogeneous responses, and evolving understanding. *Physiological Reviews*.

[19] Tisoncik J. R., Korth M. J., Simmons C. P., Farrar J., Martin T. R., Katze M. G. (2012). Into the eye of the cytokine storm. *Microbiology and Molecular Biology Reviews*.

[20] Osuchowski M. F., Welch K., Siddiqui J., Remick D. G. (2006). Circulating cytokine/inhibitor profiles reshape the understanding of the SIRS/CARS continuum in sepsis and predict mortality. *The Journal of Immunology*.

[21] Osuchowski M. F., Craciun F., Weixelbaumer K. M., Duffy E. R., Remick D. G. (2012). Sepsis chronically in MARS: systemic cytokine responses are always mixed regardless of the outcome, magnitude, or phase of sepsis. *The Journal of Immunology*.

[22] Fink M. P. (2014). Animal models of sepsis. *Virulence*.

[23] Zasada M., Kwinta P., Durlak W., Bik-Multanowski M., Madetko-Talowska A., Pietrzyk J. J. (2014). Development and maturation of the immune system in preterm neonates: results from a whole genome expression study. *BioMed Research International*.

[24] Poggi C., Bianconi T., Gozzini E., Generoso M., Dani C. (2015). Presepsin for the detection of late-onset sepsis in preterm newborns. *Pediatrics*.

[25] Zhu J. (2015). T helper 2 (Th2) cell differentiation, type 2 innate lymphoid cell (ILC2) development and regulation of interleukin-4 (IL-4) and IL-13 production. *Cytokine*.

[26] Mauri C., Menon M. (2015). The expanding family of regulatory B cells. *International Immunology*.

[27] Bateman S. L., Seed P. C. (2010). Procession to pediatric bacteremia and sepsis: covert operations and failures in diplomacy. *Pediatrics*.

[28] Drechsler S., Weixelbaumer K., Raeven P. (2012). Relationship between age/gender-induced survival changes and the magnitude of inflammatory activation and organ dysfunction in post-traumatic sepsis. *PLoS ONE*.

